# Supplementary material for: A Likelihood Approach for Real-Time Calibration of Stochastic Compartmental Epidemic Models
Source: PLoS Comput Biol. 2017 Jan 17;13(1):e1005257. doi: 10.1371/journal.pcbi.1005257 (PMC5240920; doi:10.1371/journal.pcbi.1005257)
Supplement: S1 File — (TAR.GZ) [file pcbi.1005257.s014.tar.gz › HSPH_Online-SI-Revision/EnKF07/n1-extreme/n1-extreme_EnKF2_table8.pdf]

1	newly 3 weeks spec	5.	15.35	{0., 36.}	309.	0.914018	newly 3 weeks cumul	23.	152.96	{7., 670.}	592.783	0.52968	newly 5 weeks spec	0.1	3.78	{0., 9.}	3806.	0.588802	newly 5 weeks cumul	25.	163.44	{7., 710.}	582.72	0.535419	newly 52 weeks cumul	29.	168.63	{7., 719.}	513.138	0.514518
2	newly 3 weeks spec	4.	14.14	{0., 34.}	376.5	1.03766	newly 3 weeks cumul	26.	102.42	{4., 316.}	352.077	0.519366	newly 5 weeks spec	4.	5.23	{0., 12.}	189.25	1.32961	newly 5 weeks cumul	34.	116.32	{4., 452.}	313.529	0.570155	newly 48 weeks cumul	44.	123.74	{4., 531.}	265.909	0.618728
3	newly 3 weeks spec	13.	15.19	{0., 20.}	124.077	0.686242	newly 3 weeks cumul	58.	101.97	{13., 199.}	135.914	0.380436	newly 5 weeks spec	7.	4.34	{0., 10.}	109.143	1.11597	newly 5 weeks cumul	73.	114.03	{14., 248.}	127.137	0.415181	newly 50 weeks cumul	89.	120.68	{16., 260.}	119.191	0.462859
4	newly 3 weeks spec	10.	21.55	{0., 64.}	204.9	0.66292	newly 3 weeks cumul	62.	155.94	{16., 621.}	203.645	0.396209	newly 5 weeks spec	6.	6.26	{0., 22.}	142.667	1.13287	newly 5 weeks cumul	83.	173.35	{21., 687.}	175.072	0.428726	newly 49 weeks cumul	123.	187.41	{23., 690.}	141.276	0.50723
5	newly 3 weeks spec	9.	12.38	{0., 32.}	150.	0.887952	newly 3 weeks cumul	33.	74.12	{9., 151.}	160.788	0.338831	newly 5 weeks spec	4.	7.19	{0., 18.}	217.25	1.1266	newly 5 weeks cumul	44.	90.08	{10., 208.}	152.591	0.362967	newly 42 weeks cumul	59.	125.59	{10., 410.}	179.102	0.444872
6	newly 3 weeks spec	11.	27.88	{0., 98.}	231.273	0.712626	newly 3 weeks cumul	43.	148.26	{7., 644.}	280.465	0.500482	newly 5 weeks spec	6.	11.05	{0., 37.}	189.5	0.885893	newly 5 weeks cumul	53.	177.05	{9., 737.}	271.906	0.496544	newly 47 weeks cumul	71.	195.71	{9., 747.}	228.042	0.512876
7	newly 3 weeks spec	6.	21.47	{0., 45.}	351.5	0.946766	newly 3 weeks cumul	35.	175.98	{16., 730.}	426.8	0.421902	newly 5 weeks spec	5.	6.21	{0., 11.}	166.2	1.22111	newly 5 weeks cumul	52.	192.83	{16., 730.}	307.365	0.388859	newly 51 weeks cumul	64.	205.19	{16., 730.}	267.422	0.404508
8	newly 3 weeks spec	9.	5.62	{0., 14.}	84.2222	0.843138	newly 3 weeks cumul	27.	64.59	{11., 199.}	173.37	0.337693	newly 5 weeks spec	1.	1.14	{0., 3.}	158.	0.845283	newly 5 weeks cumul	32.	68.36	{11., 205.}	153.5	0.337148	newly 45 weeks cumul	35.	69.87	{11., 205.}	143.971	0.342319
9	newly 3 weeks spec	3.	8.72	{0., 19.}	258.667	0.707099	newly 3 weeks cumul	16.	80.84	{8., 209.}	425.	0.54312	newly 5 weeks spec	3.	3.35	{0., 9.}	139.667	0.987518	newly 5 weeks cumul	22.	89.53	{8., 221.}	331.318	0.496844	newly 52 weeks cumul	25.	97.63	{8., 221.}	317.48	0.493499
10	newly 3 weeks spec	17.	27.25	{1., 99.}	145.118	0.537675	newly 3 weeks cumul	46.	144.66	{16., 480.}	248.087	0.390211	newly 5 weeks spec	5.	10.88	{0., 18.}	208.4	0.686464	newly 5 weeks cumul	57.	171.98	{25., 622.}	236.386	0.382428	newly 45 weeks cumul	77.	196.44	{29., 730.}	197.532	0.372354
11	newly 3 weeks spec	5.	28.62	{1., 98.}	517.6	0.531757	newly 3 weeks cumul	17.	158.44	{11., 586.}	844.941	0.588411	newly 5 weeks spec	0.1	7.65	{0., 29.}	7618.	1.05226	newly 5 weeks cumul	19.	181.33	{14., 700.}	867.211	0.596078	newly 51 weeks cumul	19.	189.77	{14., 751.}	911.421	0.612917
12	newly 3 weeks spec	1.	7.81	{0., 15.}	771.	0.892737	newly 3 weeks cumul	17.	130.38	{9., 375.}	685.176	0.520924	newly 5 weeks spec	2.	2.45	{0., 6.}	159.5	0.97267	newly 5 weeks cumul	22.	136.9	{10., 403.}	543.909	0.480964	newly 52 weeks cumul	22.	142.13	{12., 441.}	567.227	0.500212
13	newly 3 weeks spec	20.	8.39	{0., 22.}	76.05	1.01341	newly 3 weeks cumul	80.	197.66	{17., 692.}	190.85	0.43089	newly 5 weeks spec	17.	0.75	{0., 3.}	95.5882	1.87547	newly 5 weeks cumul	116.	200.6	{17., 693.}	133.655	0.431851	newly 45 weeks cumul	196.	201.03	{18., 693.}	94.9541	0.514841
14	newly 3 weeks spec	2.	15.11	{1., 24.}	683.5	0.712452	newly 3 weeks cumul	9.	103.88	{12., 116.}	1062.67	0.809843	newly 5 weeks spec	0.1	4.39	{0., 8.}	4374.	0.929727	newly 5 weeks cumul	9.	116.08	{12., 148.}	1197.78	0.852568	newly 49 weeks cumul	9.	123.9	{12., 179.}	1284.67	0.876228
15	newly 3 weeks spec	2.	4.87	{0., 5.}	274.5	0.950533	newly 3 weeks cumul	21.	56.05	{9., 96.}	197.857	0.289768	newly 5 weeks spec	1.	3.45	{0., 0.}	425.	0.982332	newly 5 weeks cumul	25.	62.48	{9., 107.}	192.32	0.306672	newly 44 weeks cumul	29.	66.29	{9., 112.}	182.379	0.32554
16	newly 3 weeks spec	13.	15.85	{0., 48.}	132.077	0.938288	newly 3 weeks cumul	45.	150.87	{5., 522.}	285.711	0.499772	newly 5 weeks spec	6.	3.43	{0., 9.}	90.5	1.08233	newly 5 weeks cumul	59.	160.87	{5., 534.}	227.305	0.484972	newly 51 weeks cumul	82.	175.82	{6., 545.}	179.61	0.50753
17	newly 3 weeks spec	14.	10.59	{1., 20.}	73.5	0.538421	newly 3 weeks cumul	63.	44.05	{8., 73.}	62.3651	0.404017	newly 5 weeks spec	8.	5.57	{0., 11.}	82.375	0.741503	newly 5 weeks cumul	82.	57.46	{10., 93.}	65.2927	0.425421	newly 48 weeks cumul	132.	72.2	{11., 125.}	68.697	0.530621
18	newly 3 weeks spec	15.	19.84	{1., 78.}	137.2	0.672737	newly 3 weeks cumul	37.	127.11	{11., 318.}	277.324	0.42193	newly 5 weeks spec	5.	5.97	{0., 8.}	139.8	0.942149	newly 5 weeks cumul	50.	144.53	{16., 401.}	230.46	0.409253	newly 51 weeks cumul	60.	156.97	{19., 419.}	206.783	0.399034
19	newly 3 weeks spec	10.	7.92	{0., 13.}	84.6	0.801063	newly 3 weeks cumul	28.	108.51	{16., 332.}	306.75	0.394772	newly 5 weeks spec	4.	3.62	{0., 8.}	109.5	0.951946	newly 5 weeks cumul	43.	116.96	{19., 336.}	201.442	0.340692	newly 52 weeks cumul	65.	123.05	{21., 376.}	140.908	0.351678
20	newly 3 weeks spec	4.	15.	{0., 26.}	330.	0.727889	newly 3 weeks cumul	15.	170.22	{20., 691.}	1040.67	0.722099	newly 5 weeks spec	2.	3.92	{0., 10.}	192.	0.800162	newly 5 weeks cumul	21.	180.92	{23., 716.}	769.143	0.639229	newly 45 weeks cumul	30.	187.05	{24., 716.}	534.9	0.544146
21	newly 3 weeks spec	20.	15.99	{0., 28.}	101.75	0.774934	newly 3 weeks cumul	78.	111.76	{18., 196.}	114.256	0.368548	newly 5 weeks spec	5.	5.65	{0., 11.}	135.4	0.969952	newly 5 weeks cumul	98.	126.61	{18., 215.}	108.745	0.395888	newly 48 weeks cumul	119.	141.3	{19., 238.}	106.134	0.430009
22	newly 3 weeks spec	4.	25.39	{1., 66.}	566.25	0.648222	newly 3 weeks cumul	28.	107.49	{9., 201.}	312.679	0.505191	newly 5 weeks spec	5.	11.93	{0., 20.}	215.8	0.734878	newly 5 weeks cumul	41.	137.46	{12., 288.}	268.732	0.478359	newly 49 weeks cumul	59.	163.76	{12., 445.}	218.983	0.456554
23	newly 3 weeks spec	8.	10.82	{0., 17.}	152.5	1.06555	newly 3 weeks cumul	27.	122.48	{13., 240.}	376.148	0.463335	newly 5 weeks spec	5.	4.33	{0., 6.}	138.6	1.2713	newly 5 weeks cumul	38.	134.42	{13., 409.}	286.579	0.432924	newly 51 weeks cumul	59.	143.34	{13., 414.}	195.661	0.426222
24	newly 3 weeks spec	5.	11.97	{0., 16.}	251.	0.983382	newly 3 weeks cumul	19.	111.97	{8., 392.}	511.737	0.478179	newly 5 weeks spec	2.	4.29	{0., 8.}	240.5	0.944733	newly 5 weeks cumul	25.	123.3	{9., 436.}	420.56	0.447073	newly 52 weeks cumul	25.	130.58	{9., 436.}	448.16	0.462689
25	newly 3 weeks spec	8.	10.24	{0., 19.}	163.75	1.1197	newly 3 weeks cumul	31.	97.81	{3., 324.}	275.645	0.498775	newly 5 weeks spec	8.	3.13	{0., 7.}	104.125	1.49356	newly 5 weeks cumul	48.	107.33	{3., 329.}	204.854	0.556062	newly 49 weeks cumul	114.	114.81	{3., 332.}	124.833	0.772495
26	newly 3 weeks spec	4.	18.72	{0., 18.}	429.	0.624392	newly 3 weeks cumul	13.	81.42	{8., 63.}	542.923	0.523423	newly 5 weeks spec	1.	7.74	{0., 12.}	774.	0.80206	newly 5 weeks cumul	14.	102.09	{9., 78.}	644.5	0.54356	newly 51 weeks cumul	19.	116.34	{9., 156.}	532.105	0.493774
27	newly 3 weeks spec	15.	5.64	{0., 7.}	105.333	1.41367	newly 3 weeks cumul	54.	69.16	{3., 61.}	147.333	0.684046	newly 5 weeks spec	8.	1.11	{0., 2.}	98.625	1.70588	newly 5 weeks cumul	70.	72.48	{3., 64.}	134.343	0.76406	newly 46 weeks cumul	110.	73.08	{3., 64.}	115.945	0.925047
28	newly 3 weeks spec	9.	15.78	{0., 40.}	199.333	1.16628	newly 3 weeks cumul	43.	109.23	{6., 406.}	230.349	0.581928	newly 5 weeks spec	3.	7.76	{0., 13.}	286.667	1.07221	newly 5 weeks cumul	53.	127.94	{6., 463.}	223.736	0.621311	newly 50 weeks cumul	70.	143.72	{6., 463.}	197.429	0.674396
29	newly 3 weeks spec	2.	18.49	{0., 18.}	934.5	0.953531	newly 3 weeks cumul	14.	118.27	{3., 427.}	779.929	0.52668	newly 5 weeks spec	0.1	9.92	{0., 7.}	9958.	0.570895	newly 5 weeks cumul	14.	142.21	{5., 705.}	945.357	0.534559	newly 47 weeks cumul	17.	162.49	{5., 848.}	891.824	0.54154
30	newly 3 weeks spec	2.	16.74	{0., 43.}	775.	0.726168	newly 3 weeks cumul	16.	101.86	{12., 169.}	550.125	0.505036	newly 5 weeks spec	3.	6.88	{0., 17.}	233.333	0.85843	newly 5 weeks cumul	21.	119.24	{14., 249.}	486.095	0.477492	newly 42 weeks cumul	31.	133.14	{14., 284.}	358.645	0.427863
31	newly 3 weeks spec	4.	8.9	{0., 16.}	186.	0.546071	newly 3 weeks cumul	13.	60.57	{9., 83.}	378.538	0.530565	newly 5 weeks spec	3.	3.71	{0., 5.}	152.333	0.96335	newly 5 weeks cumul	20.	69.56	{9., 119.}	265.3	0.435127	newly 51 weeks cumul	27.	78.31	{12., 140.}	212.481	0.378242
32	newly 3 weeks spec	1.	10.84	{0., 25.}	1050.	0.913348	newly 3 weeks cumul	8.	144.02	{6., 280.}	1712.	0.874301	newly 5 weeks spec	0.1	5.71	{0., 10.}	5732.	0.672339	newly 5 weeks cumul	8.	156.57	{8., 401.}	1866.88	0.913335	newly 51 weeks cumul	10.	183.02	{11., 742.}	1740.2	0.893093
33	newly 3 weeks spec	21.	18.39	{2., 39.}	96.619	0.618029	newly 3 weeks cumul	67.	79.54	{12., 148.}	95.194	0.38795	newly 5 weeks spec	11.	13.71	{0., 46.}	144.455	0.856413	newly 5 weeks cumul	89.	108.41	{14., 243.}	103.966	0.427552	newly 47 weeks cumul	124.	157.04	{15., 611.}	121.484	0.510677
34	newly 3 weeks spec	9.	14.96	{0., 12.}	182.	0.929244	newly 3 weeks cumul	31.	108.98	{8., 202.}	289.161	0.417241	newly 5 weeks spec	2.	6.	{0., 6.}	327.	0.934554	newly 5 weeks cumul	36.	124.26	{9., 211.}	285.056	0.421221	newly 50 weeks cumul	40.	131.85	{10., 212.}	272.175	0.420781
35	newly 3 weeks spec	11.	19.7	{0., 25.}	165.455	0.643924	newly 3 weeks cumul	46.	104.84	{16., 197.}	165.478	0.344508	newly 5 weeks spec	5.	9.03	{0., 14.}	191.4	0.890658	newly 5 weeks cumul	59.	126.59	{18., 203.}	155.915	0.335518	newly 51 weeks cumul	68.	153.03	{22., 236.}	166.838	0.349519
36	newly 3 weeks spec	10.	4.13	{0., 4.}	118.3	1.66263	newly 3 weeks cumul	26.	53.86	{7., 70.}	152.154	0.351993	newly 5 weeks spec	7.	2.29	{0., 1.}	116.714	1.65863	newly 5 weeks cumul	37.	59.12	{8., 75.}	123.135	0.371508	newly 48 weeks cumul	53.	66.75	{8., 75.}	117.075	0.466299
37	newly 3 weeks spec	11.	7.13	{0., 22.}	105.909	1.26612	newly 3 weeks cumul	35.	71.3	{4., 83.}	167.543	0.46043	newly 5 weeks spec	7.	1.6	{0., 6.}	93.7143	1.51503	newly 5 weeks cumul	50.	76.3	{5., 104.}	136.36	0.521686	newly 43 weeks cumul	55.	80.84	{5., 140.}	136.	0.553028
38	newly 3 weeks spec	6.	10.13	{0., 13.}	154.833	0.614938	newly 3 weeks cumul	20.	57.9	{12., 72.}	204.1	0.365789	newly 5 weeks spec	2.	4.75	{0., 6.}	255.5	0.87531	newly 5 weeks cumul	28.	69.27	{17., 90.}	163.321	0.289668	newly 48 weeks cumul	40.	81.29	{17., 94.}	132.875	0.255798
39	newly 3 weeks spec	6.	16.82	{0., 38.}	263.667	0.679243	newly 3 weeks cumul	22.	82.98	{7., 134.}	303.273	0.367845	newly 5 weeks spec	2.	7.09	{0., 10.}	355.5	0.808948	newly 5 weeks cumul	28.	100.61	{8., 202.}	291.036	0.370281	newly 51 weeks cumul	34.	114.62	{8., 346.}	275.765	0.383044
40	newly 3 weeks spec	20.	15.72	{0., 44.}	118.7	1.1033	newly 3 weeks cumul	45.	91.81	{16., 216.}	144.911	0.315407	newly 5 weeks spec	5.	7.7	{0., 25.}	199.2	1.22249	newly 5 weeks cumul	56.	111.58	{17., 287.}	151.464	0.34926	newly 49 weeks cumul	62.	133.92	{17., 426.}	174.387	0.390637
41	newly 3 weeks spec	17.	9.84	{0., 14.}	122.471	1.57563	newly 3 weeks cumul	35.	75.83	{6., 128.}	184.771	0.446548	newly 5 weeks spec	9.	7.24	{0., 6.}	150.	1.59348	newly 5 weeks cumul	50.	91.85	{8., 232.}	168.7	0.507774	newly 44 weeks cumul	61.	120.92	{9., 256.}	188.82	0.553343
42	newly 3 weeks spec	1.	17.14	{2., 33.}	1618.	1.01685	newly 3 weeks cumul	20.	87.21	{16., 164.}	348.35	0.543503	newly 5 weeks spec	1.	9.1	{0., 17.}	844.	0.69403	newly 5 weeks cumul	23.	108.64	{19., 239.}	383.826	0.565194	newly 52 weeks cumul	25.	142.87	{23., 276.}	481.4	0.594346
43	newly 3 weeks spec	13.	17.44	{0., 18.}	152.	0.920882	newly 3 weeks cumul	49.	93.87	{6., 317.}	165.367	0.455962	newly 5 weeks spec	1.	6.47	{0., 8.}	647.	0.826982	newly 5 weeks cumul	57.	111.6	{6., 331.}	170.175	0.465099	newly 51 weeks cumul	68.	125.27	{8., 337.}	162.456	0.490895
44	newly 3 weeks spec	6.	18.99	{0., 44.}	265.833	0.550696	newly 3 weeks cumul	30.	98.92	{15., 206.}	248.933	0.401888	newly 5 weeks spec	3.	9.45	{0., 19.}	300.333	0.769481	newly 5 weeks cumul	42.	121.67	{18., 276.}	213.071	0.37087	newly 49 weeks cumul	55.	150.49	{18., 323.}	204.018	0.372047
45	newly 3 weeks spec	9.	19.13	{0., 57.}	203.	0.774638	newly 3 weeks cumul	41.	130.46	{9., 254.}	267.951	0.453798	newly 5 weeks spec	11.	5.56	{0., 15.}	102.727	1.30342	newly 5 weeks cumul	64.	146.33	{9., 299.}	201.234	0.505171	newly 52 weeks cumul	78.	154.15	{9., 327.}	180.397	0.543519
46	newly 3 weeks spec	12.	10.83	{0., 25.}	98.5833	0.655734	newly 3 weeks cumul	35.	87.9	{11., 104.}	187.143	0.31611	newly 5 weeks spec	5.	4.1	{0., 10.}	98.8	0.883303	newly 5 weeks cumul	52.	99.02	{12., 138.}	142.462	0.33587	newly 49 weeks cumul	69.	109.06	{12., 168.}	125.333	0.376898
47	newly 3 weeks spec	16.	11.31	{0., 16.}	109.688	1.11827	newly 3 weeks cumul	65.	105.07	{4., 173.}	143.092	0.514947	newly 5 weeks spec	11.	3.75	{0., 8.}	98.6364	1.44693	newly 5 weeks cumul	87.	115.16	{4., 179.}	126.828	0.56267	newly 50 weeks cumul	118.	126.99	{4., 210.}	115.958	0.638527
48	newly 3 weeks spec	17.	11.97	{0., 27.}	121.824	1.40247	newly 3 weeks cumul	49.	135.81	{6., 377.}	268.469	0.651522	newly 5 weeks spec	7.	3.83	{0., 4.}	129.	1.57411	newly 5 weeks cumul	64.	146.49	{6., 379.}	228.391	0.69218	newly 50 weeks cumul	85.	156.38	{7., 486.}	191.859	0.745187
49	newly 3 weeks spec	8.	23.23	{0., 72.}	260.625	0.64269	newly 3 weeks cumul	31.	118.17	{11., 227.}	306.613	0.433107	newly 5 weeks spec	3.	9.44	{0., 32.}	305.333	0.86637	newly 5 weeks cumul	39.	142.85	{13., 327.}	295.308	0.432733	newly 52 weeks cumul	53.	160.67	{15., 504.}	242.132	0.420705
50	newly 3 weeks spec	6.	15.16	{0., 56.}	254.	0.934658	newly 3 weeks cumul	38.	174.16	{12., 457.}	393.474	0.467481	newly 5 weeks spec	6.	1.84	{0., 6.}	96.	1.39305	newly 5 weeks cumul	47.	181.11	{15., 464.}	328.021	0.455987	newly 50 weeks cumul	63.	182.82	{15., 519.}	246.254	0.453599
